# *SARPPIC*: Exploiting COVID-19 Contact Tracing Recommendation through Social Awareness

**DOI:** 10.1155/2020/3460130

**Published:** 2020-11-10

**Authors:** Nana Yaw Asabere, Amevi Acakpovi, Emmanuel Kwaku Ofori, Wisdom Torgby, Marcellinus Kuuboore, Gare Lawson, Edward Adjaloko

**Affiliations:** ^1^Department of Computer Science, Accra Technical University, Ghana; ^2^Department of Electrical/Electronic Engineering, Accra Technical University, Ghana; ^3^Department of Chemical Pathology, University of Ghana, Ghana; ^4^Department of Information Technology Studies, University of Professional Studies, Ghana

## Abstract

Globally, the current coronavirus disease 2019 (COVID-19) pandemic is resulting in high fatality rates. Consequently, the prevention of further transmission is very vital. Until vaccines are widely available, the only available infection prevention methods include the following: contact tracing, case isolation and quarantine, social (physical) distancing, and hygiene measures (washing of hands with soap and water and using alcohol-based hand sanitizers). Contact tracing, which is key in preventing the spread of COVID-19, refers to the process of finding unreported people who maybe infected by using a verified case to trace back possible infections of contacts. Consequently, the wide and fast spread of COVID-19 requires computational approaches which utilize innovative algorithms that build a memory of proximity contacts of cases that are positive. In this paper, a recommender algorithm called socially aware recommendation of people probably infected with COVID-19 (*SARPPIC*) is proposed. *SARPPIC* initially utilizes betweenness centrality in a social network to measure the number of target contact points (nodes/users) who have come into contact with an infected contact point (COVID-19 patient). Then, using contact durations and contact frequencies, tie strengths of the same contact points above are also computed. Finally, the above algorithmic computations are hybridized through profile integration to generate results for effective contact tracing recommendations of possible COVID-19-infected patients who will require testing in a healthcare facility. Benchmarking experimental results in the paper demonstrate that, using two interconnected relevant real-world datasets, *SARPPIC* outperforms other relevant methods in terms of suitable evaluation metrics such as precision, recall, and F-measure.

## 1. Introduction

Coronavirus disease (COVID-19), which originated in December 2019 from the city of Wuhan in China, is caused by severe acute respiratory syndrome–coronavirus 2 (SARS-CoV-2) [1]. Globally, COVID-19 has clearly shown its potential of high fatality rates. A global response pertaining to effective health systems and delivery is extremely imperative and vital. Over one hundred (100) countries worldwide have been hit severely by COVID-19 and are currently awaiting reliable and sustainable vaccines [1–3].

Consequently, until innovative vaccines are made widely available, the only existing infection prevention methods are contact tracing, case isolation and quarantine, social (physical) distancing, and hygiene measures such a washing hands with soap and water and using alcohol-based hand sanitizers [1–3]. Globally, as different states and local governments seek a way out of lockdowns that have brought their economies to a near standstill, “contact tracing” has made its way into everyday conversations as well. COVID-19 contact tracing is similar to detective work. Trained staff conduct interviews with people who have been diagnosed with COVID-19 to verify who they may have recently been in contact with. Then, once those who have been in contact with the COVID-19 patient is known, the trained staff inform them that they may have been exposed and encourage them to quarantine themselves to prevent spreading of the disease any further.

This process is very laborious. Interviewing COVID-19 patients and reaching out to dozens of contacts takes time. For this reason, contact tracing works best when there are low levels of infection in a community. However, many high-level communities worldwide are currently affected by COVID-19. Consequently, contact tracing methods need to be improved through technology (computerization and automation). The use of a contact tracing application (app) which is designed using computational algorithms for building a memory of proximity contacts would be adequate to stop the epidemic to some extent [1, 2]. Such an application can immediately notify and recommend contacts of positive cases.

Recommender systems have become very popular due to the fact that they can help users to find items of interest (such as movies, books, and music) in order to cope with the information overload (big data) problem [4]. In the past decade, many researchers have worked to develop recommender systems which involve people to people recommendations; some of these include [5–8]. With reference to people-to-people recommendations, the current issue of contact tracing in COVID-19 can also be tackled using recommender systems.

This paper proposes a recommender algorithm called socially aware recommendation of people probably infected with COVID-19 (*SARPPIC*). *SARPPIC* utilizes the computations of between centrality and social ties [9] as contact tracing entities in a social network to generate recommendations regarding possibly infected COVID-19 patients. The major contributions of this paper are summarized as follows:

(i) Betweenness centrality and accurate tie strength computations are applied in a social graph network for predicting people who may be infected with COVID-19.

(ii) A recommendation method which exploits and hybridizes information regarding high betweenness and tie strength to generate people-to-people recommendations is proposed.

(iii) Using appropriate evaluation metrics, benchmarking experiments were conducted on two interconnected relevant real-world datasets, to verify the effectiveness of the proposed recommendation method.

The rest of this paper is structured as follows. Section 2 discusses related studies pertaining to this paper. Section 3 outlines the details of the proposed *SARPPIC* method. Section 4 elaborates on the performance evaluation and further discusses the results achieved. Finally, Section 5 concludes the paper.

## 2. Related Studies

This section presents related studies and literature pertaining to the study. Related studies in the paper focus on the following: (i) people-to-people recommender systems, (ii) social recommendations through tie strength and betweenness centrality, and (iii) computational/automation methods in COVID-19 contact tracing.

### 2.1. People-to-People Recommender Systems

People-to-people prediction and recommendation has recently become an imperative task in many online social networks. In recommender systems, traditional collaborative filtering (CF) approaches are popular for effectively predicting user preferences for items. However, in online social networks, people have a dual role as both “users” and “items,” e.g., both initiating and receiving contacts [8]. The main objective of people-to-people recommender systems is to generate meaningful social suggestions to users [8, 10]. Some related studies regarding people-to-people recommendation include the following: Cai et al. [8] proposed a recommendation model called *SocialCollab* which fully captures the bilateral role of user interactions within a social network and formulates CF methods to enable people-to-people recommendation. In their recommendation model, users can be similar to other users in two ways—either having similar “attractiveness” for the users who contact them or having similar “taste” for the users they contact. Similarly, Tsai and Brusilovsky [10] proposed an innovative method which integrates a global search result using a personalized people-to-people recommender system. Their method employs the user identity as a query keyword and processes the search results through five different customized parsers. In relation to the problem involving CF over-recommending popular items, Krzywick et al. [11] addressed this problem in the context of people-to-people recommendations. Similarly, Bourke et al. [12] examined the practice of leveraging a user's social graph in order to generate people recommendations. Using various neighbourhood selection strategies, they investigated user satisfaction and the level of perceived trust in the recommendations received. On a large commercial online dating site, Wobcke et al. [7] reported on the successful deployment of a people-to-people recommender system. The deployment was the result of thorough evaluation and an online trial of a number of methods. Xia et al. [5] and Asabere et al. [6] similarly proposed linear hybrid recommender algorithms which employ accurate prediction of tie strengths and personality to generate conference participant (people) recommendations at a smart conference.

### 2.2. Social Recommendations through Betweenness Centrality and Tie Strength

In a social network, betweenness centrality is a measure of the influence of a vertex over the flow of information between every pair of vertices under the postulation that information primarily flows over the shortest paths between them [13]. Various researchers have applied betweenness centrality in their recommendation approaches. Noteworthy research work regarding such methods includes the following: Samad et al. [14] employed textual and topological similarity measures for citation recommendation of relevant/important research papers and then computed betweenness centrality measures to generate recommendations of important papers for researchers. Similarly, by exploiting betweenness centrality, Sie et al. [15] proposed a novel tool which employs similar mindedness to recommend potential co-authors. Similar to [14, 15], Christensen and Schiaffino [16] applied a betweenness centrality approach to propose a social recommendation method which involved group modeling.

Mahyar et al. [17] utilized a betweenness centrality concept to propose a novel method which improves recommendation accuracy in accordance to the most central users who are designated as group heads. Pucci et al. [18] utilized betweenness centrality to depict a random-walk-based scoring recommender algorithm, which recommends top-rank items to possibly interested users. Sulieman et al. [19] expounded on a recommendation method between items using a mixed approach of social network analysis and content as well as CF. From a root item, a relationship's graph is generated and used to extract network metrics through betweenness and closeness centrality measures. Souza et al. [20] proposed an algorithmic method which recommends items based on social network centrality and semantic relevance.

Tie strength or social ties usually refer to the social interactions between individuals. A meaningful social relationship such as friendship between two individuals represents the existence or not of ties [9, 21, 22]. Social ties can be categorized into strong ties (e.g., family members or trusted friends) that share relevant information with a huge overlap. In contrast, weak ties (e.g., acquaintances) share more diverse and new information [21]. Quite recently, the application of social ties in recommender systems has been exploited by various researchers. Some of such related studies are presented below.

Xia et al. [23] and Asabere et al. [24] similarly addressed the recommendation of presentation sessions at smart conferences to attendees using their social ties. They proposed a venue recommender algorithm called socially aware recommendation of venues and environments (SARVE). In relation to the research paper recommendation, Asabere et al. [25] and Xia et al. [26] improved the social awareness of attendees in a smart conference by proposing an innovative folksonomy-based paper recommender algorithm, namely, socially aware recommendation of scholarly papers (SARSP).

Using Facebook data for the use case of online news with 193 participants, Oechslein and Hess [21] developed a research model and tested it in an online experiment. Their proposed structural equation model results showed that strong tie relationships have positive impacts on recommendation value. Similarly, using a strong concept of social ties, Jang et al. [27] proposed a system which detects and analyzes the behaviors of group-level socializing to support ex post facto and real-time social applications deployed in real social event situations.

### 2.3. Computational/Automation Methods in COVID-19 Contact Tracing

In relation to the current and global COVID-19 pandemic and the high proportion of transmissions from presymptomatic individuals, controlling the epidemic through manual contact tracing is infeasible. Quite recently, a number of contact tracing technological solutions have been introduced. For example, from the very early stage of the epidemic, China and South Korea commenced tracing COVID-19 victims and their contacts through facial recognition technologies as well as smartphones [1]. Consequently, the extensive and rapid spread of COVID-19 requires computational approaches which utilize innovative algorithmic and mathematical methods. Very recently, some authors have developed algorithms and models in this regard. Significant among these are as follows:

Ferretti et al. [1] developed an algorithm which involves a mathematical model that encompasses the memory of proximity contacts and immediately notifies contacts of positive cases through a contact tracing app. Abler et al. [28] discussed the implementation of a contact tracing app based on Bluetooth low energy technology between two people to control COVID-19. Similar to [1, 28], Yasaka et al. [29] proposed an anonymized graph of interpersonal interactions to conduct a novel form of contact tracing and further developed a proof-of-concept smartphone app which implements their approach. Additionally, they developed a computer simulation model that validates their proposed method. Drew et al. [30] developed algorithms and modeled a COVID-19 Symptom Tracker mobile application. Their mobile application offers clinical outcomes, herald symptoms, geographical hot spots, and data on risk factors.

Current trends of the computational COVID-19 contact tracing research enumerated above show that there is lack of social properties inclusion regarding people infected with COVID-19 and their respective contacts in a social network. Due to the fact that COVID-19 is a social issue, social properties are very important factors worth considering [9, 22]. The notion of social properties attracts substantial interest initially from the social and behavioral communities, as well data mining communities and network communities [9, 22]. Different from the research work enumerated, this paper proposes a graph-based social recommendation method which utilizes betweenness centrality and social ties as social properties of probable COVID-19 contacts to develop a recommender algorithm (*SARPPIC*). Benchmarking experimental results below have verified the effectiveness of the proposed method.

## 3. Proposed Solution—SARPPIC Method

**Figure 1:**
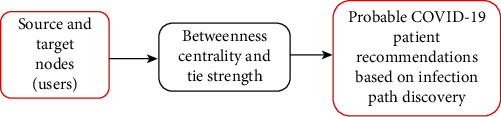
Fundamental recommendation procedure of SARPPIC.

**Figure 2:**
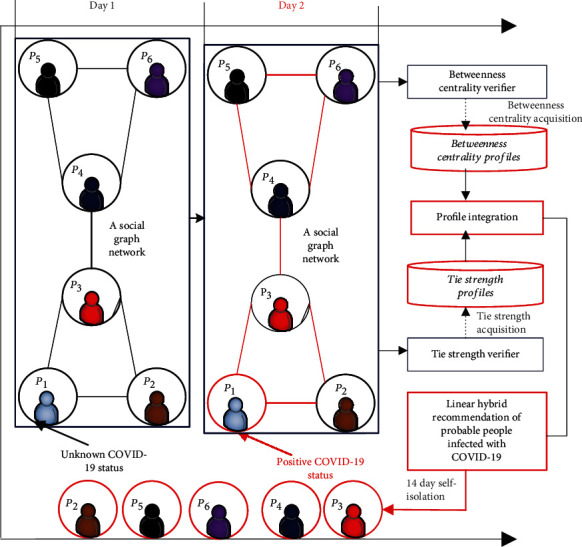
SARPPIC recommendation model.

This section presents the framework and proposed solution of the *SARPPIC* recommendation method.  Figure 1 depicts the fundamental recommendation procedure of *SARPPIC*. In relation to infection path discovery, Figure 2 shows that, through the *betweenness centrality verifier*, *SARPPIC* initially verifies and computes betweenness centralities of contact points in the social graph network. Additionally, *SARPPIC* utilizes the *tie strength verifier* to compute tie strength profiles of the contact points through their contact durations and contact frequencies for onward linear hybrid recommendation of probable people infected with COVID-19. Further elaboration on the proposed *SARPPIC* recommendation model is presented below.

### 3.1. COVID-19 Infection Path Discovery

Central to the proposed *SARPPIC* recommendation method is a data structure which is referred to as a social graph. Generally, social networks illustrate the small world ideology that node encounters are adequate to build a connected relationship graph. A social graph is an appropriate tool which represents the relational structure of social networks in a natural manner. In a social graph, vertices (nodes) indicate human individuals, and edges (links) indicate social relationships between individuals [9, 22].

In the proposed method, the social graph consists of directed edges (links), which represent transmission vectors between contact points (nodes). The concept of betweenness centrality is utilized in a defined social graph shown in Figure 2. Betweenness centrality specifies the betweenness of a vertex in a network, and it indicates the extent to which a vertex lies on the shortest paths between pairs of other vertices. In many real-world situations such as the current COVID-19 pandemic, it has quite a significant role. As shown in Equation (1), the Betweenness Centrality (*BC*) of a vertex *v*, i.e., BC(*v*) for any graph is defined as follows:

(1) $$\begin{array}{c} \text{BC}\left(v\right)=\underset{a,b\in V}{\sum }\left(\frac{\sigma_{ab}\left(v\right)}{\sigma_{ab}}\right), \end{array}$$

where *σ*_*ab*_(*v*)is the total number of shortest paths between nodes *a* and *b* that pass through *v*, and *σ*_*ab*_ is the total number of shortest paths between nodes *a* and *b*. The proposed *SARPPIC* recommendation method computes the BC of node *P*_3_ as tabulated in Table 1.

In the social graph in Figure 2, each contact point denotes a physical interaction between two or more individuals at a specific time and place, during which microorganisms could potentially be transmitted from one individual to others. Each contact point in the social graph can be classified in one of the two states: *positive status* or *unknown status*. A positive status represents a contact point which has been identified as having COVID-19 infection, while an unknown status denotes contact points who are not yet infected by COVID-19.

With reference to Figure 2, using the simple data structure of the social graph, possible transmission paths can be determined for any given target contact point. A possible transmission path is defined as a path from a positive status node which could be carrying microorganisms from a reported point of exposure to a given target node with an unknown status. A demonstration of a simple transmission graph is provided in Figure 2.

According to Figure 2, there are six contact points and they are connected. Figure 2 shows that on day 1, contact point *P*_1_ has an unknown status. However, on day 2, *P*_1_ has a positive status. There is no shortest path of *P*_3_ involving the paths of the following: *P*_1_ to *P*_2_, *P*_4_ to *P*_5_, *P*_4_ to *P*_6_, and *P*_5_ to *P*_6_; hence, *σ*_*ab*_(*v*) = 0 in all these cases. However, movement between any contact points (e.g., *P*_1_ to *P*_4_) in the network as depicted in Table 1 utilizes the shortest path of *P*_3_; therefore, *σ*_*ab*_(*v*) = 1 in all these cases.

Therefore, as shown in Table 1, BC for node *P*_3_ in Figure 2 is the summation of all values which resulted in 1. BC for node *P*_3_ is therefore equal to 6. Furthermore, due to the fact that nodes *P*_3_ and *P*_4_ are on the same level in the network, the BC for node *P*_4_ will also be 6.  Algorithm 1 shows the computation of highest BC. A high betweenness count for an infected contact point indicates that the contact point holds authority over other contact points in the social graph network. Consequently, as illustrated in Figure 2, due to the fact that on day 2, *P*_1_ has a positive status; if *P*_1_ transmits to *P*_3_, the high centrality of *P*_3_ paves the way for all other contact points (*P*_4_, *P*_5_, and *P*_6_) to also be infected with COVID-19.

### 3.2. Social Ties/Tie Strength of Contact Points

Existing research literature above has shown evidence that in a social network, the relationship, connection, and influence of users enhance reliability, effectiveness, and productivity. Furthermore, recommender systems research has provided evidence that the application of social elements and attributes improves recommendation quality and accuracy by avoiding cold start as well as data sparsity problems [4–6]. In the proposed *SARPPIC* recommendation method, another common social property called social ties/tie strength is utilized. Computations of social ties/tie strength are done through contact duration and contact frequency of contact points [5, 6, 9, 21–25].

Generally, the social ties/tie strength of two users in a social network are computed to verify the extent of their relationship and the influence they have on each other. Equation (2) is used to compute the tie strengths of contact points in the social network graph. As shown in Figure 2, these computations are utilized as a strategy to establish the strength of relationship for profile integration with prior betweenness centrality computations, in order to generate effective linear hybrid recommendations of people probably infected with COVID-19.

(2) $$\begin{array}{c} \text{Tie\_strengt}{\text{h}}_{P3,P2}\left(t+∆t\right)=\text{tie\_strengt}{\text{h}}_{P3,P2}\left(t-∆t\right)+\left(1-\delta \right)\times \text{tie\_strengt}{\text{h}}_{P3,P2}\left(t\right). \end{array}$$

In Equation (2) show above, tie_strength_*P*1,*P*2_(*t* − Δ*t*) and tie_strength_*P*1,*P*2_(*t*) are the past and present social ties/tie strengths between *P*_3_ and *P*_2_, where *P*_3_ is the contact point with the highest BC, and *P*_2_ is a target node (contact point). *δ* is a parameter that resolves the influence proportion of the present and past social ties, and Δ*t* is the time frame used to compute the social ties/tie strength between *P*_3_ and *P*_2_.

### 3.3. SARPPIC Algorithm and Linear Hybrid Recommendation

**Figure 3:**
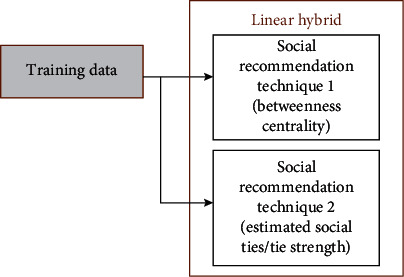
Training phase process in SARPPIC.

**Figure 4:**
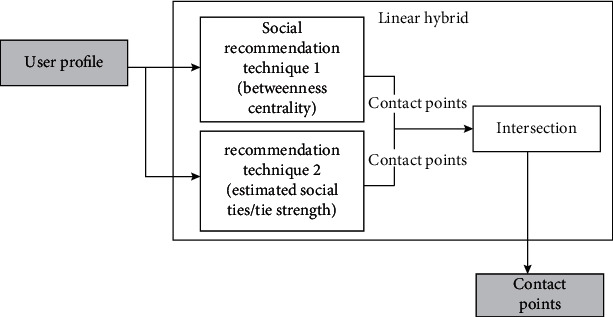
Contact point profile modeling in SARPPIC.

Figure 3 demonstrates the training phase of *SARPPIC*. In Figure 3, each individual social recommendation technique processes the training data. After the training phase, Figure 4 shows how the modeling of user profiles relating to contact points for test users are generated. Therefore, these recommendation techniques jointly propose contact points who have common intersections of user profiles, in terms of the contact point with the highest BC and corresponding tie strengths. Contact point generation is essential in verifying people who will be considered in the linear hybrid recommendation.

**Figure 5:**
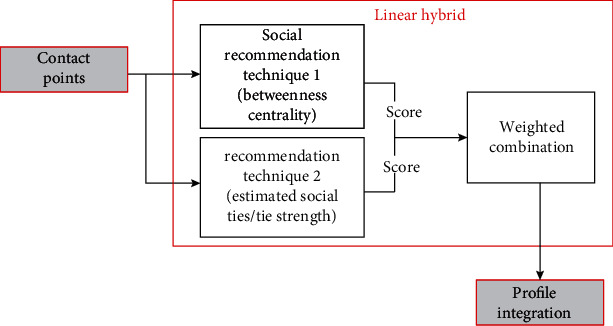
Profile integration process in SARPPIC.

As illustrated in Figure 5, the contact points are then sorted out through their combined weighted score, and high-valued profile integrations validate a top linear hybrid recommendation. As explained above, in the experimentation procedure, computations of betweenness centrality and social ties/tie strength of contact points are incorporated using Equations (1) and (2), respectively. The incorporation of the results for Equations (1) and (2) is linearly hybridized using Equation (3) below. The proposed *SARPPIC* recommendation method therefore improves recommendation accuracy and also enhances the socially aware recommendation for probable people with COVID-19 in a social network graph.

(3) $$\begin{array}{c} \text{PI}\left(P_{3},P_{2}\right)=\text{tie\_strengt}{\text{h}}_{P3,P2}\left(t+\Delta t\right)+\text{BC}\left(v\right). \end{array}$$

Through profile integration, Equation (3) merges the results of Equations (1) and (2) to finally compute the linear hybridization of *P*_3_ and *P*_2_, in terms of betweenness centrality and tie strength of contact points. Furthermore, in the experimentation procedure, *α* is utilized in Equation (4) below to set a threshold for to Equation (3), so that linear hybrid recommendations related to COVID-19 contact points can effectively be determined and generated.

(4) $$\begin{array}{c} \text{PI}\left(P_{3},P_{2}\right)\geq \alpha . \end{array}$$

The proposed *SARPPIC* algorithm (Algorithm 2) declares relevant variables in steps 2-4; the computations and hybridizations of BC and tie strength in relation to contact points are shown in steps 6-12. The generation of linear hybrid recommendations of probable people infected with COVID-19 is illustrated in the steps 13-17, which are the final steps of the proposed *SARPPIC* algorithm.

## 4. Performance Evaluation of SARPPIC

This section presents a sequence of scientific benchmarking experiments to validate the performance of *SARPPIC*. The scientific experimentation procedure compared *SARPPIC* to similar algorithmic methods in [8, 28], respectively, represented as COV-1 and COV-2. The benchmarking experiments were done using computers with the following specifications: Microsoft Windows 64-Bit, 8 GB RAM, 500 HDD, and 3.90 GHz dual intel core processors.

### 4.1. Datasets and Evaluation Metrics

In order to achieve favourable and reliable experimental results, during the scientific experimentation process, two real-world datasets were interconnected and utilized, namely, HEXACO-60 dataset which is available in IEEE Data Port (doi:10.21227/phht-pn81) and the ATU dataset in SARVE-2 [31].

As shown in Table 2, the HEXACO-60 dataset contains 60 contact points and a total of 249 betweenness centrality data. In Table 2, the contact points with the highest betweenness centrality initially required for experimentation in accordance to the *SARPPIC* algorithm are C02, C08, and C14 (26), C38, C32, and C26 (19), H06, H12, and H16 (31), and X40, X34, and X28 (23).

In relation to social tie data, Tables 3 and 4 illustrate the details of past and present tie strength data in [31]. The interconnected datasets were divided into 80% and 20% for the training and test sets, respectively.

During the experimentation process, four commonly used evaluation metrics were employed: (a) precision (*P*), which is the ratio of the number of probable people (contact point) infected with COVID-19 in the *top-N* recommendation list denoted as Num(*N*, *d*) to the length of the same list denoted as Num(*N*); (b) recall (*R*), which is the ratio of the number of probable people (contact points) infected with COVID-19 in the *top-N* recommendation list represented as Num(*N*, *d*) to the total number of all contact points in the social network graph represented Num(*d*); (c) F1 is a harmonic mean of precision and recall; and (d) AM is the arithmetic mean of precision and recall. The equations used for computations of these utilized metrics are illustrated in Equations (5), (6), (7) and (8).

(5) $$\begin{array}{c} P=\frac{\text{Num}\left(N,d\right)}{\text{Num}\left(N\right)}, \end{array}$$

(6) $$\begin{array}{c} R=\frac{\text{Num}\left(N,d\right)}{\text{Num}\left(d\right)}, \end{array}$$

(7) $$\begin{array}{c} \text{F}1=\frac{2\times P\times R}{P+R}, \end{array}$$

(8) $$\begin{array}{c} \text{AM}=\frac{1}{2}\left(P+R\right). \end{array}$$

### 4.2. Baseline Methods and Experimental Parameters

As stated in Section 4 above, the benchmarking experiments involved the performance comparison of *SARPPIC* to the methods in [28, 29] denoted as COV-1 and COV-2, respectively. These methods were selected due to their high relevance and similarity to *SARPPIC* in terms of COVID-19 contact tracing.

The benchmarking experiments are aimed at alleviating data sparsity and cold-start challenges [4–6]. In order to substantiate the experimental results, the following questions required answers:

1. In comparison to COV-1 and COV-2, what was the overall performance of *SARPPIC*?
2. In comparison to COV-1 and COV-2, how did *SARPPIC* perform in terms of cold-start and data sparsity reduction?

In relation to experimental parameters, the computations of the profile integration coefficients ranged from 6.0 to 11.0. Consequently, profile integration coefficients (*N*) ranging from 6.0 to 11.0 were utilized for testing and the rest of the computed data for training. During the experimentation process, it was noticed that *N* results from between 8.0 and 11.0 were more reliable and favourable for effective generation of recommendations involving probable people infected with COVID-19. With reference to Equation (4), the above range (0.8 to 11.0) was therefore used as a threshold to substantiate recommendation quality and accuracy.

### 4.3. Experimental Results and Analysis

**Figure 6:**
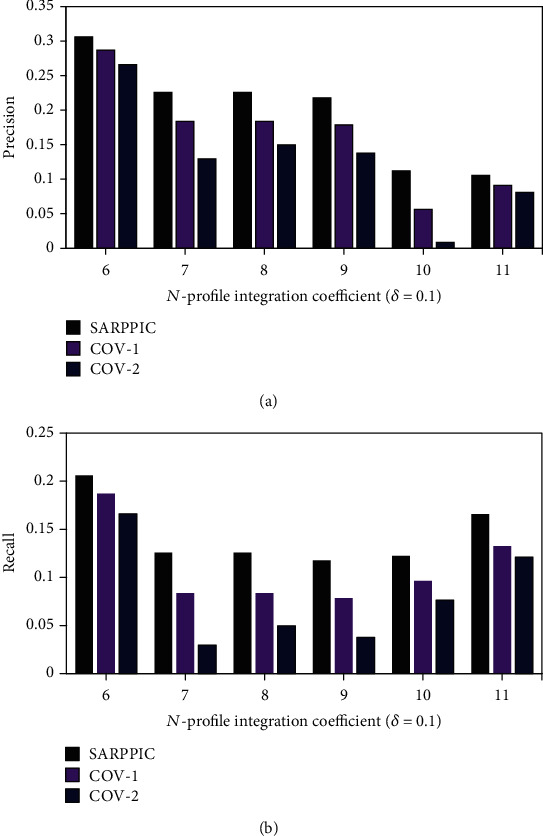
(a) Precision performance results on datasets (*δ* = 0.1); (b) recall performance results on datasets (*δ* = 0.1).

**Figure 7:**
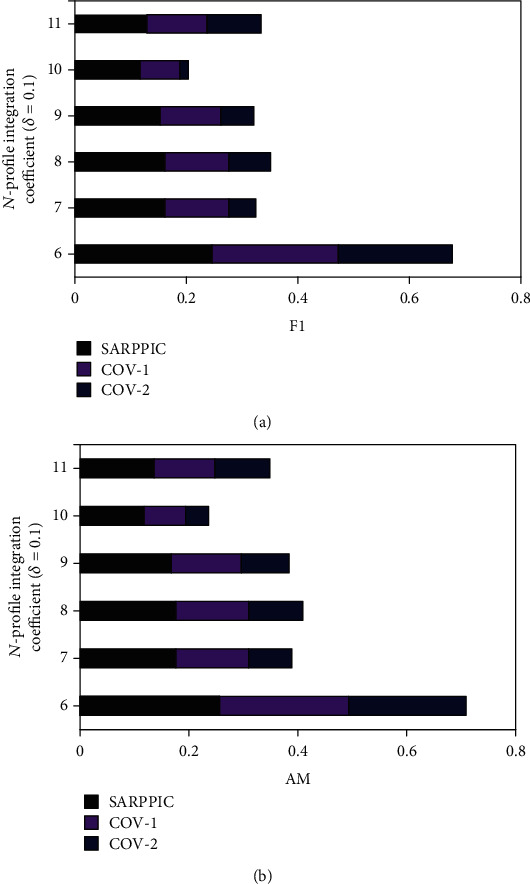
(a) F1 performance results on datasets (*δ* = 0.1); (b) AM performance results on dataset (*δ* = 0.1).

In this section, experimental results and analysis are presented. Figures 6 and 7 show the comparison results of *SARPPIC*, COV-1, and COV-2 (when *δ* equals 0.1) on the HEXACO-60-ATU dataset. As can be verified from this figure, *SARPPIC* achieves much larger values of precision, recall, F1, and AM than COV-1 and COV-2 for *different top-N* recommendations in terms of *N*-profile integration coefficients (6.0 to 11.0). In particular, *SARPPIC* significantly achieves high improvement (approximately 10% in precision—Figure 6(a); 16% in recall—Figure 6(b); 49% in F1—Figure 7(a); and 13% in AM—Figure 7(b)), when *N* is 11.

**Figure 8:**
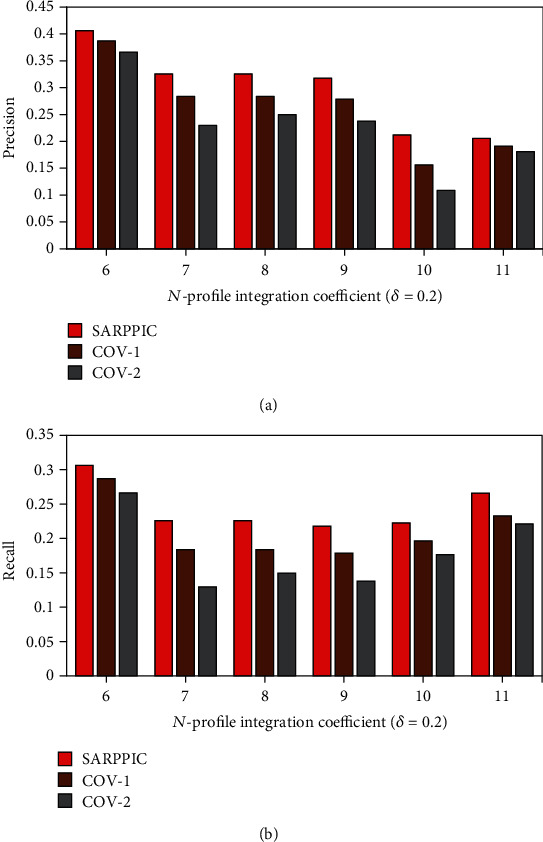
(a) Precision performance results on dataset (*δ* = 0.2); (b) recall performance results on dataset (*δ* = 0.2).

**Figure 9:**
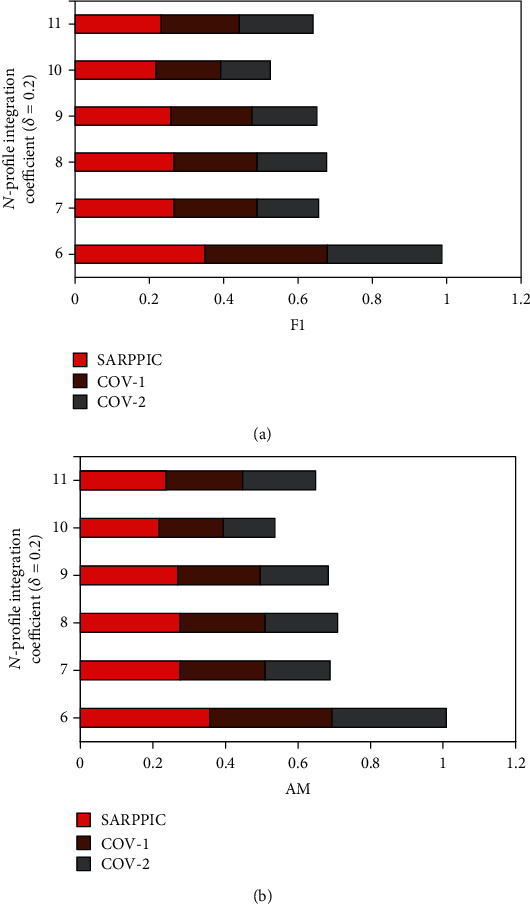
(a) F1 performance results on dataset (*δ* = 0.2); (b) AM performance results on dataset (*δ* = 0.2).

In addition, further comparison of experimental results of *SARPPIC*, COV-1, and COV-2 (when *δ* equals 0.2) on the HEXACO-60-ATU dataset is shown in Figures 8 and 9. From these figures, it can also see that as the value of *N* is increasing, *SARPPIC* always achieves large values of precision, recall, F1, and AM than COV-1 and COV-2 (approximately 20% in precision—Figure 8(a); 26% in recall—Figure 8(b); 79% in F1—Figure 9(a); and 23% in AM—Figure 9(b)), when *N* is 11. As shown in Tables 5 and 6, the experimental process authenticates that the AM results achieved for *SARPPIC* are the higher comparison to that of F1 (harmonic mean), which accordingly substantiates that AM should always be higher than F1 regarding the retrieval effectiveness of a recommender system/algorithm [32].

These experimental results on the HEXACO-60-ATU dataset demonstrate that *SARPPIC* significantly outperforms COV-1 and COV-2 in terms of the four evaluation metrics. Furthermore, this indicates that the hybridized profile integration of high BC and tie strength is able to help generate more accurate social recommendations relating to probable people infected with COVID-19.

In summary, Figures 6–9 as well as Tables 5 and 6 show that *SARPPIC* reliably attained more promising results in all the utilized evaluation metrics. Furthermore, Table 7 depicts the comparison of *SARPPIC* to similar algorithms wih relevant explanations in each case. These observations coroborate that *SARPPIC* is more suitable, robust, and reduces cold-start and data sparsity challenges due to its capability to utilize profile integration of BC and tie strength. Furthermore, in relation to prediction/recommendation accuracy, the experimental results also depict the importance of social properties in comparison to traditional CF for the generation of effective people-to-people recommendations of probable people with COVID-19.

## 5. Discussion and Concluding Remarks

Due to the current global COVID-19 pandemic, which is causing high fatality rates, a novel people-to-people (social) recommendation method called *SARPPIC* has been proposed. *SARPPIC* hybridizes two main social properties, namely, betweenness centrality and social ties/tie strength. *SARPPIC* initially applies betweenness centrality to compute the highest betweenness of contact point transmissions in the social graph network, which have transmission authority over other contact points. Secondly, the tie strengths of identified contact points with high betweenness and other target contact points are computed. The *SARPPIC* recommender algorithm then hybridizes the above computations involving high betweenness and tie strengths to generate recommendations of probable people infected with COVID-19.

Extensive benchmarking experiments on two interconnected real-world datasets have been conducted to validate the effectiveness of *SARPPIC* in terms of people-to-people recommendation for contact points in a social graph network with high betweenness centrality and strong social ties. Experimental results clearly illustrate the effectiveness of SARPPIC in comparison to other contemporary methods in terms of appropriate evaluation metrics. The consideration and utilization of other social properties such as closeness centrality, degree centrality, and social neighbours as well as the concept of personality in the current COVID-19 pandemic need to be explored. Future work will focus on these research issues.

## Figures and Tables

**Algorithm 1:**
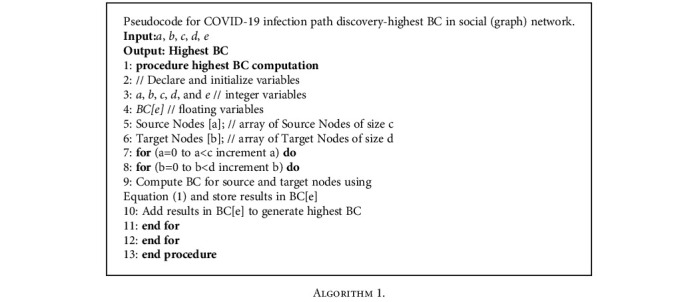
Algorithm 1.

**Algorithm 2:**
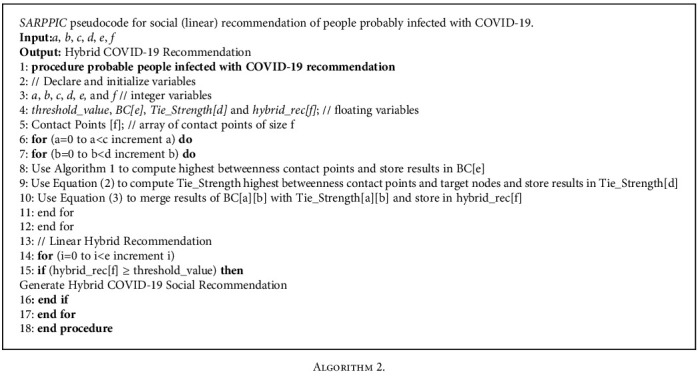
Algorithm 2.

**Table 1:** BC computations of contact points in Figure 2.

Source node	Target node	*σ*_*ab*_	*σ*_*ab*_(*v*)	$\frac{\sigma_{ab}\left(v\right)}{\sigma_{ab}}$
*P*_1_	*P*_2_	1	0	0
*P*_1_	*P*_4_	1	1	1
*P*_1_	*P*_5_	1	1	1
*P*_1_	*P*_6_	1	1	1
*P*_2_	*P*_4_	1	1	1
*P*_2_	*P*_5_	1	1	1
*P*_2_	*P*_6_	1	1	1
*P*_4_	*P*_5_	1	0	0
*P*_4_	*P*_6_	1	0	0
*P*_5_	*P*_6_	1	0	0

**Table 2:** Dataset: centrality indices for the HEXACO-60 network.

Nodes	Betweenness
H06, H12, H16	5
*H24, H30, H36*	31
H42, H48	14
H64, H60	0
E05, E11	6
E17, E23	2
E29, E35, E41	3
E47, E53, E59	17
X58, X52, X46	11
*X40, X34, X28*	23
X04, X10	7
X16, X22	12
A9, A15	5
A21, A57, A33	5
A27, A51, A03	14
A39, A45	5
C56, C50, C44	7
*C02, C08, C14*	26
C20	5
*C38, C32, C26*	19
O01, O55, 025	14
O31, O37, O43	5
O13, O49	10
O19, O07	3

**Table 3:** ATU dataset—contact frequency trends.

Past tie strength data		Present tie strength data	
Contact frequency	Number of contact points	Contact frequency	Number of contact points
1	263	1	438
2	1246	2	1127
3	669	3	805
4	477	4	485
5	291	5	245
6	243	6	134
7	108	7	63

**Table 4:** ATU dataset—contact duration trends.

Past social tie data		Present social tie data	
Contact duration	Number of contact points	Contact duration	Number of contact points
5	126	5	129
10	370	10	438
15	165	15	154
20	405	20	420
25	299	25	244
30	471	30	432
35	229	35	223
40	301	40	298
45	124	45	119
50	254	50	239
55	61	55	62
60	181	60	182
65	145	65	161
70	104	70	104
75	1	75	1
80	61	80	61

**Table 5:** *P*, *R*, F1, and AM performance on dataset (*δ* = 0.1).

Method	Highest *N*	Precision	Recall	F1	AM
COV-1	11.0	0.09	0.13	0.10	0.11
*SARPPIC*	11.0	0.10	0.16	0.12	0.13
COV-2	11.0	0.08	0.12	0.09	0.10

**Table 6:** *P*, *R*, F1, and AM performance on dataset (*δ* = 0.2).

Method	Highest *N*	Precision	Recall	F1	AM
COV-1	11.0	0.19	0.23	0.20	0.21
*SARPPIC*	11.0	0.20	0.26	0.23	0.24
COV-2	11.0	0.18	0.22	0.19	0.20

**Table 7:** Proposed algorithm comparison with similar algorithms (advantages and disadvantages).

Criteria	Algorithms	
	*SARPPIC*	COV-1 and COV-2
Recommendation entities	Profile integration of social properties, i.e., BC and tie strength/social ties as entities for recommendation which is very appropriate for COVID-19 contact tracing.	These algorithms do not utilize social properties, i.e., BC and tie strength as entities for recommendation.

Cold-start and data sparsity challenges	Reduction of cold-start and data sparsity challenges due to its (*SARPPIC*'*s*) capability of utilizing social properties, i.e., tie strength/social ties (through contact durations and frequencies) and BC (through shortest paths).	These algorithms utilize traditional collaborative filtering (CF) methods as entities and therefore the effect of cold start and data sparsity is not as minimal as compared to that of *SARPPIC* due to less social property inclusion.

Algorithm performance in terms of evaluation metrics	In terms of utilized evaluation metrics, namely, precision, recall, F1, and AM (Tables 5 and 6), *SARPPIC* outperforms COV-1 and COV-2 in relation to effective generation of people-to-people recommendations (COVID-19 patients) due to robustness, suitability, and effective social property inclusion for efficient contact tracing.	In terms of utilized evaluation metrics, namely, precision, recall, F1, and AM (Tables 5 and 6), COV-1 and COV-2 do not perform to the level of *SARPPIC* in relation to people-to-people recommendations (COVID-19 patients) due to nonutilization of social properties for efficient contact tracing.

## Data Availability

The (data type) data used to support the findings of this study are included within the article. Two real-world datasets were interconnected and utilized, namely, HEXACO-60 dataset which is available in IEEE Data Port at doi:10.21227/phht-pn81 and the ATU dataset in SARVE-2 [31] available at doi:10.1109/TETC.2018.2854718.

## Supplementary Materials

Supplementary Materials The supplementary material of this paper elaborates on the dataset which was utilized for the experimentation process. A dataset description of the HEXACO-60 and ATU datasets, which can be accessed through relevant links, is appropriately provided in the supplementary material.
