# Supplementary material for: SARPPIC: Exploiting COVID-19 Contact Tracing Recommendation through Social Awareness
Source: Comput Math Methods Med. 2020 Nov 10;2020:3460130. doi: 10.1155/2020/3460130 (PMC7667519; doi:10.1155/2020/3460130)
Supplement: Supplementary Materials — The supplementary material of this paper elaborates on the dataset which was utilized for the experimentation process. A dataset description of the HEXACO-60 and ATU datasets, which can be accessed through relevant links, is appropriately provided in the supplementary material. [file 3460130.f1.pdf]

AUTHORS/RESEARCHERS:

Nana Yaw Asabere[1], Amevi Acakpovi[2], Emmanuel Kwaku Ofori[3], Wisdom Torgby[1], Marcellinus Kuuboore[4], Gare Lawson[1] and Edward Adjaloko[1]

Department of Computer Science, Accra Technical University, Accra-Ghana[1]

Department of Electrical/Electronic Engineering, Accra Technical University, Accra-Ghana[2]

Department of Chemical Pathology, University of Ghana, Accra-Ghana[3]

Department of Information Technology Studies, University of Professional Studies, Accra-Ghana[4]

Correspondence E-Mail: yawasbere2011@gmail.com; yawasabere2005@yahoo.com

RESEARCH ARTICLE: SARPPIC: Exploiting COVID-19 Contact Tracing Recommendation Through Social Awareness

DATASET: Graphical-Representation-of-the-Network-of-the-HEXACO-60-Items

We utilized and interconnected two real-world data sets namely: HEXACO-60 Dataset which is available in IEEE Data Port at: http://dx.doi.org/10.21227/phht-pn81 and the ATU Dataset in SARVE-2 [1] available at DOI: 10.1109/TETC.2018.2854718 .

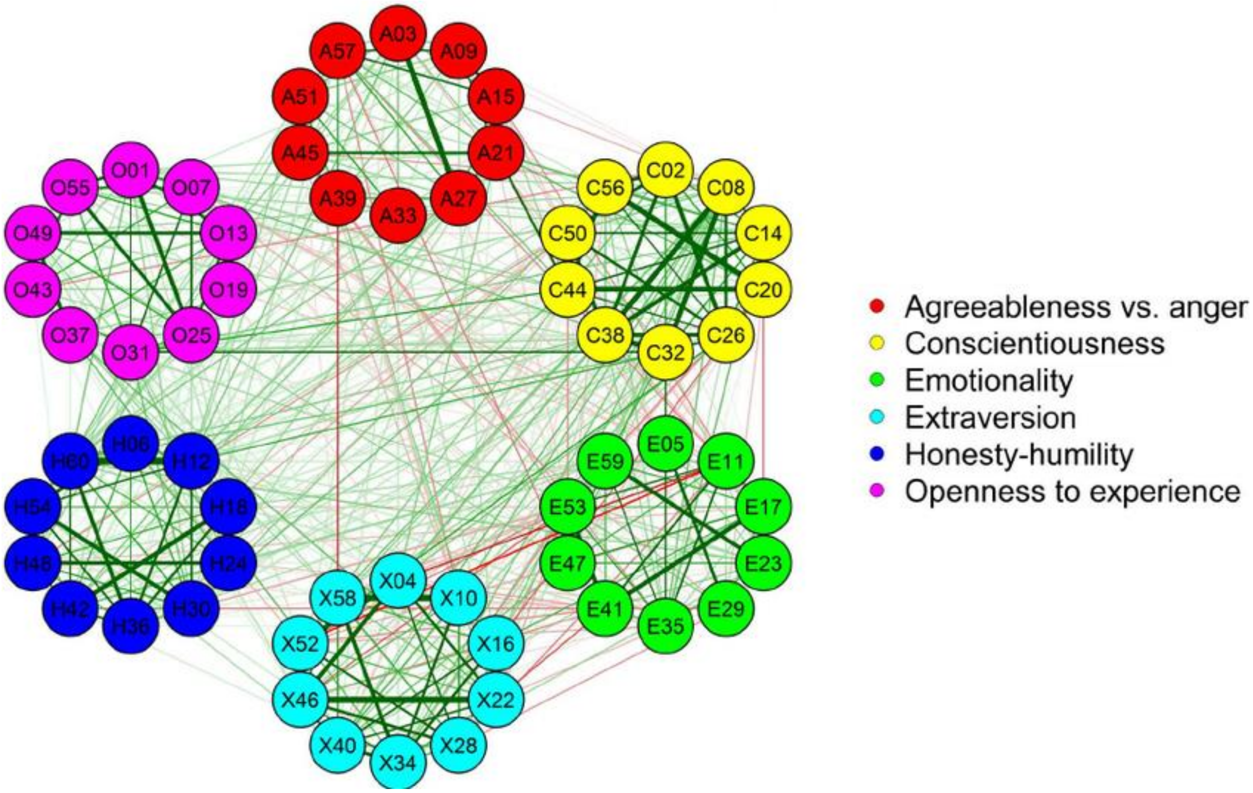

As shown above, the HEXACO-60 dataset contains 60 contact points and a total of 249 betweenness

centrality data. The contact points with the highest betweenness centrality initially required for experimentation in accordance to our *SARPPIC* algorithm are C02, C08 and C14 (26), C38, C32, and C26 (19), H06, H12 and H16 (31), and X40, X34 and X28 (23).

As stated in [1], data was gathered from students (contact points in *SARPPIC*) at Accra Technical University in Higher National Diploma (HND) Marketing (*n*=2864), HND Computer Science (*n*=720) and HND Building Technology (*n*=812). Therefore, the ATU dataset utilized in this paper contains a total of 4396 users (2421 males representing 55.07% and 1975 females representing 44.93%). The present and past social ties of the ATU Data set are shown in the Tables below. Additionally, we have attached an excel file of all the data collected and how computations were done using appropriate formulas.

TABLE IV

ATU DATASET- CONTACT DURATION TRENDS

Past Social Tie Data		Present Social Tie Data	
Contact Duration	Number of Attendees	Contact Duration	Number of Attendees
5	126	5	129
10	370	10	438
15	165	15	154
20	405	20	420
25	299	25	244
30	471	30	432
35	229	35	223
40	301	40	298
45	124	45	119
50	254	50	239
55	61	55	62
60	181	60	182
65	145	65	161
70	104	70	104
75	1	75	1
80	61	80	61

TABLE I

ATU DATASET- CONTACT FREQUENCY TRENDS

Past Tie Strength Data		Present Tie Strength Data	
Contact Frequency	Number of Contact Points	Contact Frequency	Number of Contact Points
1	263	1	438
2	1246	2	1127
3	669	3	805
4	477	4	485
5	291	5	245
6	243	6	134
7	108	7	63

REFERENCE

1. N. Y. Asabere, B. Xu, A. Acakpovi, and N. Deonauth, “SARVE-2: exploiting social venue recommendation in the context of smart conferences,” *IEEE Transactions on Emerging Topics in Computing*, 2018. DOI: 10.1109/TETC.2018.2854718
